# Computation of Entropy Production in Stratified Flames Based on Chemistry Tabulation and an Eulerian Transported Probability Density Function Approach

**DOI:** 10.3390/e24050615

**Published:** 2022-04-28

**Authors:** Louis Dressler, Hendrik Nicolai, Senda Agrebi, Florian Ries, Amsini Sadiki

**Affiliations:** 1 Department of Mechanical Engineering, Reactive Flows and Diagnostics, Technical University of Darmstadt, Otto-Berndt-Str. 3, 64287 Darmstadt, Germany; agrebi@rsm.tu-darmstadt.de (S.A.); ries@ekt.tu-darmstadt.de (F.R.); sadiki@ekt.tu-darmstadt.de (A.S.); 2 Department of Mechanical Engineering, Simulations of Reactive Thermo-Fluid Systems, Technical University of Darmstadt, Otto-Berndt-Str. 2, 64287 Darmstadt, Germany; nicolai@stfs.tu-darmstadt.de

**Keywords:** entropy generation, combustion, large eddy simulation, flamelet generated manifold, eulerian stochastic fields

## Abstract

This contribution presents a straightforward strategy to investigate the entropy production in stratified premixed flames. The modeling approach is grounded on a chemistry tabulation strategy, large eddy simulation, and the Eulerian stochastic field method. This enables a combination of a detailed representation of the chemistry with an advanced model for the turbulence chemistry interaction, which is crucial to compute the various sources of exergy losses in combustion systems. First, using detailed reaction kinetic reference simulations in a simplified laminar stratified premixed flame, it is demonstrated that the tabulated chemistry is a suitable approach to compute the various sources of irreversibilities. Thereafter, the effects of the operating conditions on the entropy production are investigated. For this purpose, two operating conditions of the Darmstadt stratified burner with varying levels of shear have been considered. The investigations reveal that the contribution to the entropy production through mixing emerging from the chemical reaction is much larger than the one caused by the stratification. Moreover, it is shown that a stronger shear, realized through a larger Reynolds number, yields higher entropy production through heat, mixing and viscous dissipation and reduces the share by chemical reaction to the total entropy generated.

## 1. Introduction

Recently, the analysis of entropy generation has emerged as an important tool to evaluate the efficiency of energy conversion systems, as it allows to estimate the amount of work - the exergy—that can be extracted from such systems. Thereby, the second law of thermodynamics accounts for the irreversibilities taking place in such energy conversion systems and enables the quantification of occurring exergy losses. Depending on the system considered, many sources of irreversibilities can be encountered. These include mechanical dissipation, heat conduction, diffusion, chemical reactions, phase change, among others. Focusing on fluid flows, such irreversibilities provoke a degradation of the available energy into internal energy in the working fluid leading to an increase of the system entropy [1,2,3,4]. Using the entropy generation analysis allows, therefore, to identify causes of inefficiencies in systems along with the significance of irreversibilities generated by each specific process in the thermo-fluid system under consideration. Subsequently, it aids to delimit the evolution of these processes, and at the same time, gives access to the control and possible minimization of the irreversibilities. Such analysis has been especially beneficial in accessing the thermodynamic efficiency of heat exchangers [3], power plants [5], or internal combustion engines [6,7] and providing guidelines on how these systems may be improved. A comprehensive approach to determine the overall exergy destruction is to compare the exergy entering and the exergy exiting the system [1]. While this approach is straightforward, it gives only little insight into the ongoing processes themselves and on their respective entropy production rates [8]. However, looking at combustion systems, many different physical processes (mass- and heat transport, chemical reaction, turbulence) occur, more or less simultaneously and each process is linked to a certain amount of exergy loss [1]. Therefore, for such complex systems, a more detailed approach is needed. A summary of possible methods to estimate the exergy losses in reactive systems is given in Som and Datta [1], where the authors point out that second-law-based investigations have mainly been performed using low fidelity modeling strategies. This is mainly due to the fact that the usage of more sophisticated and detailed models is rather restricted because of the inherently connected high computational costs. This constraint leads to contributions either concentrating on laminar flames [9,10,11], on simplified turbulent configurations using direct numerical simulation (DNS) [12], or on turbulent flames using reduced-order models [11,13,14]. However, the processes generally occurring in combustion systems include exergy losses that vary spatially and temporally. This makes large eddy simulation (LES) a favorable tool to investigate the entropy generation in such complex systems [15]. As pointed out in [15], the contributions in this field are still scarce. To mention are the works of Safari et al. [15,16], which are both dedicated to entropy source term closures in the context of combustion LES. Here, the closure is achieved by transporting the filtered subgrid scalar probability density function (PDF) through a particle-based Monte Carlo approach. The framework is applied in [15] to the well-known Sandia flame D benchmark case [17]. Also related is the contribution of Agrebi et al. [18] where different approaches for computing the entropy production terms are compared for the same flame. The approach adopted is based on the Eulerian Stochastic Field (ESF) method [19,20] coupled to a chemistry tabulation strategy [21]. These investigations were dedicated to flames burning predominantly in a diffusion mode.

This contribution aims at shedding light on the unexplored area of representing irreversibilities in turbulent stratified premixed flames [22,23] in the context of the LES and tabulated chemistry. To this end, two operating conditions of the Darmstadt Stratified Burner [24] are investigated using LES. With respect to numerical investigations, many contributions have been dedicated to this configuration [25,26,27,28,29], which is closely related to the extensive validation data set provided [24,25,30]. However, while the previous numerical studies mainly focused on the validation of the modeling strategy, the present work looks from the exergy-loss-perspective onto this configuration. An overview of the recent advances made in simulations of turbulent stratified premixed combustion can be found in the extensive review paper by Lipatnikov [23].

The main objectives of this paper can be summarized as follows: (1) Demonstrate the suitability of tabulated chemistry for computing the sources of irreversibilities in stratified premixed flames; (2) propose a new approach to compute the subgrid contributions to the arising entropy production terms based on the ESF method; (3) analyze the impact of stratification in the premixed flames on the entropy production contribution of the different processes, (4) investigate the effect of a stronger shear, realized through a higher Reynolds number, on these entropy production terms.

The rest of this paper consists of four sections. The section following this brief introduction familiarizes the reader with the modeling framework adopted. Thereafter, the investigated configuration and numerical setup are outlined in Section 3. Next, the results are presented and discussed in Section 4. Finally, Section 5 summarizes the main outcomes.

## 2. Methods

This section introduces the modeling strategies used. First, the filtered transport equations for reactive fluid flows are introduced in the context of tabulated chemistry. This subsection is then followed by a part dedicated to modeling the turbulence chemistry interaction. Here, the Eulerian stochastic fields method is briefly outlined. Thereafter, the computation of the entropy source terms in reactive flows and the closure to compute their unresolved contributions are presented.

### 2.1. LES and Tabulated Chemistry

Large eddy simulation is performed in this work, where only large structures are resolved and residual (or subgrid) contributions must be modeled. This concept is reflected in the filtered transport equations for mass and momentum

(1) $$\begin{array}{cc} \frac{\partial \bar{\rho }}{\partial t}+\frac{\partial }{\partial x_{i}}\left(\bar{\rho }{\tilde{u}}_{i}\right) & =0, \end{array}$$

(2) $$\begin{array}{cc} \frac{\partial \bar{\rho }{\tilde{u}}_{i}}{\partial t}+\frac{\partial }{\partial x_{j}}\left(\bar{\rho }{\tilde{u}}_{i}{\tilde{u}}_{j}\right) & =-\frac{\partial \bar{p}}{\partial x_{i}}+\frac{\partial }{\partial x_{j}}\left(\bar{\mu }\left(\frac{\partial {\tilde{u}}_{i}}{\partial x_{j}}+\frac{\partial {\tilde{u}}_{j}}{\partial x_{i}}-\frac{2}{3}\frac{\partial {\tilde{u}}_{k}}{\partial x_{k}}\delta_{ij}\right)\right)-\frac{\partial }{\partial x_{j}}\left(\bar{\rho }\tau_{ij}^{sgs}\right), \end{array}$$

where ρ is the density, $u_{i}$ the *i*th component of the Cartesian velocity, μ the dynamic viscosity of the fluid, and *p* the dynamic pressure. Here, $\tau_{ij}^{sgs}$ represents the (deviatoric part of ) the subgrid-scale (sgs) momentum transport, which requires modeling. Filtered and Favre-filtered variables are represented by $\bar{\left(\;\cdot \;\right)}$ and $\tilde{\left(\;\cdot \;\right)}$, respectively. Considering reactive systems, these equations are generally complemented by a set of transport equations for each species involved and the enthalpy. However, the direct application of detailed kinetics is currently restricted to simple cases due to the high computational costs linked to (1) the transport of all chemical species and (2) the stiff coupling of these equations emerging from the reaction kinetics. Tabulated chemistry approaches enable a detailed representation of the chemistry at affordable computational costs. The idea can be summarized as follows: (1) Compute the chemistry in a preprocessing step. (2) Map the results onto a reduced set of variables to create a thermochemical lookup table. (3) Within the LES, solve a transport equation for each mapping variable and retrieve the thermochemical state through a table lookup at runtime. Several implementations of this concept have been proposed [21,31,32,33,34]. In this work, the flamelet generated manifold (FGM) approach [33] is adopted, where the thermochemical state is mapped on the two table controlling variables of mixture fraction *Z* and progress variable PV, to represent the effects of mixing and chemical reaction. In this work, the lookup table consists of one-dimensional premixed flamelets, each computed at a different equivalence ratio and assuming a unity Lewis number for all species. This assumption is justified by an effective Lewis number close to one for Air-$\begin{array}{c} errortypeceCH4 \end{array}$ mixtures [35] as well as by previous investigations, where this assumption has proven adequate [25,27,29]. Similarly to [25,27], the mixture fraction is expressed as the sum of carbon and hydrogen element mass fractions in the mixture. The progress variable is defined through the $\begin{array}{c} errortypeceCO2 \end{array}$ mass fraction [25,27]. Equations (1) and () are therefore extended by

(3) $$\begin{array}{cc} \frac{\partial \bar{\rho }\tilde{Z}}{\partial t}+\frac{\partial }{\partial x_{i}}\left(\bar{\rho }\tilde{Z}{\tilde{u}}_{i}\right) & =\frac{\partial }{\partial x_{i}}\left(\frac{\bar{\mu }}{Sc}\frac{\partial \tilde{Z}}{\partial x_{i}}-\bar{\rho }\tau_{Z}^{sgs}\right), \end{array}$$

(4) $$\begin{array}{cc} \frac{\partial \bar{\rho }\tilde{PV}}{\partial t}+\frac{\partial }{\partial x_{i}}\left(\bar{\rho }\tilde{PV}{\tilde{u}}_{i}\right) & =\frac{\partial }{\partial x_{i}}\left(\frac{\bar{\mu }}{Sc}\frac{\partial \tilde{PV}}{\partial x_{i}}-\bar{\rho }\tau_{PV}^{sgs}\right)+\bar{\rho }{\tilde{\dot{\omega }}}_{PV}, \end{array}$$

where the modeled subgrid-scale fluxes for mixture fraction and progress variable are represented by $\tau_{Z}^{sgs}$ and $\tau_{PV}^{sgs}$, and ${\tilde{\dot{\omega }}}_{PV}$ denotes the filtered progress variable reaction source term. The Schmidt number Sc is set to a value of 0.7. The unclosed terms $\tau_{i}^{sgs}$ in the Equations (2)–(4) are modeled through the σ-eddy-viscosity model [36] introducing the subgrid-scale viscosity $\nu_{sgs}$. The Reynolds analogy is used in the case of *Z* and PV. The subgrid-scale fluxes read

(5) $$\tau_{ij}^{sgs}=-2\nu_{sgs}\left({\tilde{S}}_{ij}-\frac{1}{3}{\tilde{S}}_{kk}\delta_{ij}\right),\;\tau_{Z}^{sgs}=\frac{\nu_{sgs}}{Sc_{sgs}}\frac{\partial \tilde{Z}}{\partial x_{i}},\;\tau_{PV}^{sgs}=\frac{\nu_{sgs}}{Sc_{sgs}}\frac{\partial \tilde{PV}}{\partial x_{i}},$$

with the strain rate tensor ${\tilde{S}}_{ij}=\left(\frac{\partial u_{i}}{\partial x_{j}}+\frac{\partial u_{j}}{\partial x_{i}}\right)$. The introduced subgrid-scale viscosity is given as [36]

(6) $$\nu_{sgs}={\left(C_{\sigma }\Delta \right)}^{2}\frac{\sigma_{3}\left(\sigma_{1}-\sigma_{2}\right)\left(\sigma_{2}-\sigma_{3}\right)}{\sigma_{1}^{2}},$$

with the model constant $C_{\sigma }=1.7$, Δ the filter width, and $\sigma_{i}$ the *i*th singular value of the resolved velocity gradient. For the scalar subgrid fluxes, a constant subgridscale Schmidt number is introduced $Sc_{sgs}=0.7$ [25,27,37]. The proper representation of the filtered progress variable reaction source term is addressed in the next section.

### 2.2. Modeling of the Turbulence Chemistry Interaction

One issue that arises in LES is the proper representation of the interaction of the chemistry with the turbulent structures, i.e., resolved and unresolved parts. To tackle this problem, a multitude of different approaches have been introduced in the context of LES [38,39,40]. These approaches range from purely advective methods [41], artificial flame thickening [42,43] and flame filtering [44] to statistical methods [45]. The latter can be subdivided into presumed and transported PDF approaches. In this work, a transported PDF approach is adopted, namely the Eulerian Stochastic Field method [19,20,46]. In contrary to presumed approaches where the filtered subgrid PDF (FDF) is presumed *a-priori*, the ESF method approximates the FDF at runtime by an ensemble of $N_{s}$ stochastic fields for each of the table controlling variables. The method requires solving a stochastic differential equation for each of the so-called Eulerian stochastic fields $\xi_{\alpha }$, representing a δ-peak in Z−PV composition space. The stochastic fields evolve according to [19,20]

(7) $$\begin{array}{cc} d(\bar{\rho }\xi_{\alpha }^{n})= & -\frac{\partial }{\partial x_{j}}\left(\bar{\rho }\xi_{\alpha }^{n}u_{j}\right)dt+\frac{\partial }{\partial x_{i}}\left[\left(\frac{\bar{\mu }}{Sc}+\frac{\mu_{sgs}}{Sc_{sgs}}\right)\frac{\partial \xi_{\alpha }^{n}}{\partial x_{i}}\right]dt+\bar{\rho }{\dot{\omega }}_{\alpha }^{n}\left(\phi^{n}\right)dt\\ & +\frac{\bar{\rho }}{\tau_{t}}\left(\xi_{\alpha }^{n}-{\tilde{\phi }}_{\alpha }\right)dt+\bar{\rho }\sqrt{\frac{2}{\bar{\rho }}\frac{\mu_{sgs}}{Sc_{sgs}}}\frac{\partial \xi_{\alpha }^{n}}{\partial x_{j}}dW_{j}^{n} \end{array},$$

where α={Z,PV} and $n=(1,2,\ldots ,N_{s})$. The last term on the right-hand side (RHS) is the stochastic contribution to the equation arising from the unresolved turbulent structures in the presence of scalar gradients for the individual field. This is different from the classical modeled subgrid-scale flux, which is applied to the whole filtered density function. The term $dW^{n}=\eta \sqrt{\Delta t}$ is the increment-vector of a stochastic Wiener process, which is constant in space but different for each field. Accordingly, $dW_{j}^{n}$ denotes its *j*th component. The Wiener process is a normally distributed random walk with a mean of zero and a variance of the time step size Δt. Various approaches to represent sub-filter micro-mixing exist, as the Euclidean minimum spanning tree (EMST) [47], the Fokker-Planck (FP) [48] or the interaction by exchange with the mean (IEM) [48,49], also known as linear mean square estimation closure (LMSE) [50,51] model. In this work, mixing at the unresolved level is described using the IEM model. It is worth noting that the IEM is a deterministic model which does not contain PDF shape features. Its initial PDF shape will be maintained and cannot relax to a Gaussian distribution. In this model, the change of the composition is directly related to the mean without being affected by the other stochastic fields. In this respect, it does not fulfill the requirement of localness in the composition space. Important to mention is the strong IEM limitation due to the mixing rate which is the same for all the components, implying that the difference in diffusion is not considered. Nevertheless, this model has proven good performance in LES [52,53,54,55,56]. For this model, the micro-mixing time scale $\tau_{t}$ is utilized and expressed as [57]

(8) $$\tau_{t}={\left(C\frac{\nu +\nu_{sgs}}{\Delta^{2}}\right)}^{-1},$$

with the micromixing constant C=2[37,53,58,59] and the filter width $\Delta ={\left(\Delta_{x}\Delta_{y}\Delta_{z}\right)}^{1/3}$. The stochastic fields are not only to obtain the moments of the marginal FDFs, but also provide a closure for the reaction source terms

(9) $${\tilde{\dot{\omega }}}_{PV}=\frac{1}{N_{s}}\sum_{n=1}^{N_{s}}{\dot{\omega }}_{PV}\left(\xi_{Z}^{n},\xi_{PV}^{n}\right)$$

As previously outlined, the stochastic increments should be sampled from a normal distribution. However, for a low finite number of stochastic fields, sampling the components of the vector from a normal distribution will rarely match the zero mean and ΔT variance constraint. To bypass this issue, a weak first-order approximation is applied. The increments are sampled from a dichotomic distribution {1,−1}[60]. Here, the correct mean and variance are enforced by introducing complementary increments $\eta_{i}^{j+N_{s}/2}=-\eta_{i}^{j}$ for the second half of the stochastic increments. This set of vectors is then randomly shuffled to avoid any correlation between the fields [59]. For this purpose, an even number of stochastic fields is required. For the solution of a coupled set of partial differential equations with stochastic components, additional challenges arise. The main problem in the present context is the density derivative in the continuity equation. The stochastic fluctuations in the density fields directly impact the pressure solution, which is tightly interwoven with the momentum transport, thus making the solution procedure prone to numerical instabilities [61]. In the context of stochastic particle-based methods, these density fluctuations are usually treated through an additional enthalpy equation in Eulerian form, for which the source term is obtained from the stochastic particles (instead of directly computing the density from the particles) [62,63,64]. In the present work, a similar approach as proposed by Prasad [65] has been applied and adapted to the tabulated chemistry framework. The procedure introduces so-called auxiliary moments, which are less sensitive to stochastic fluctuations. Subsequently, these auxiliary control variables are used to obtain the filtered density and viscosity, which are used consistently in all equations solved. The procedure allows to perform stable simulations using low numbers of stochastic fields. In the present work, either 4, 8, or 16 stochastic fields are used to investigate the sensitivity of the results with respect to the number of fields. For more information, the reader is referred to [54,58].

### 2.3. Computation of the Entropy Generation Source Terms

The exergy analysis is centered around the computation of exergy losses, i.e. its destruction, occurring in a given system in order to improve the system performance. These exergy losses can be expressed through the sum of the entropy production sources rates $\sum_{i}\Pi_{i}$ and the ambient temperature $T_{0}$ as follows [66,67]:

(10) $${\dot{Ex}}_{loss}=T_{0}\sum_{i}\Pi_{i}.$$

The processes generally occurring in combustion systems include exergy losses, which vary spatially and temporally. This can be deducted by considering the transport equation for the Favre filtered entropy $\tilde{s}$ assuming a unity Lewis number for all species [15,68]

(11) $$\begin{array}{cc} \frac{\partial \bar{\rho }\tilde{s}}{\partial t}+\frac{\partial }{\partial x_{i}}\left(\bar{\rho }\tilde{u_{i}s}\right) & -\frac{\partial }{\partial x_{i}}\left(\bar{\rho }D\frac{\partial \tilde{s}}{\partial x_{i}}\right)=\\ \; & \underset{{\bar{\Pi }}_{V}}{\underset{︸}{\bar{\frac{1}{T}\tau_{ij}\frac{\partial u_{i}}{\partial x_{j}}}}}+\underset{{\bar{\Pi }}_{Q}}{\underset{︸}{\bar{\frac{\lambda }{T^{2}}\frac{\partial T}{\partial x_{i}}\frac{\partial T}{\partial x_{i}}}}}+\underset{{\bar{\Pi }}_{D}}{\underset{︸}{\bar{\frac{\lambda }{c_{p}}\sum_{k}^{N}\frac{R_{k}}{Y_{k}}\frac{\partial Y_{k}}{\partial x_{i}}\frac{\partial Y_{k}}{\partial x_{i}}}}}+\underset{{\bar{\Pi }}_{CR}}{\underset{︸}{\bar{\frac{1}{T}\sum_{k}^{N_{s}}\mu_{k}{\dot{\omega }}_{k}}}}. \end{array}$$

Here, λ is the thermal conductivity, $c_{p}$ the heat capacity at constant pressure, $R_{k}$ and $Y_{k}$ are the specific gas constant and mass fraction of the *k*th species, respectively. The chemical potential and source term of the *k*th species are denoted by $\mu_{k}$ and ${\dot{\omega }}_{k}$. The RHS of Equation (11) consists of four entropy production terms. These represent the filtered entropy production by viscous dissipation ${\bar{\Pi }}_{V}$, heat transport ${\bar{\Pi }}_{Q}$(annihilation of temperature inhomogeneities), mixing ${\bar{\Pi }}_{D}$(annihilation of mixture inhomogeneities) and chemical reaction ${\bar{\Pi }}_{CR}$. From Equation (11), it can be deduced that the filtering operation prevents a straightforward computation of the production terms. The various production terms must be modeled based on known quantities. For this purpose, the entropy source terms are decomposed into resolved and subgrid contributions

(12) $$\Pi_{i}=\Pi_{i}^{res}+\Pi_{i}^{sgs},\;i=\left\{V,Q,D,CR\right\}.$$

For the entropy production through viscous dissipation, the approach from Ries et al. [69] is applied. This term is expressed by

(13) $${\bar{\Pi }}_{V}=\bar{\frac{1}{T}\tau_{ij}\frac{\partial u_{i}}{\partial x_{j}}}=\underset{\Pi_{V}^{res}}{\underset{︸}{\left(\tilde{\frac{1}{T}}\right)\bar{\mu }\left(\frac{\partial {\tilde{u}}_{i}}{\partial x_{j}}+\frac{\partial {\tilde{u}}_{j}}{\partial x_{i}}\right)\frac{\partial {\tilde{u}}_{i}}{\partial x_{i}}}}+\underset{\Pi_{V}^{sgs}}{\underset{︸}{\left(\tilde{\frac{1}{T}}\right)\bar{\rho }\varepsilon_{k_{sgs}}}},$$

where the turbulent kinetic energy dissipation rate $\varepsilon_{k_{sgs}}$ has been introduced. Mere dimensional considerations lead to [69,70]

(14) $$\varepsilon_{k_{sgs}}=\frac{1}{\Delta^{4}C_{S}^{4}}\nu_{sgs}^{3},$$

where $C_{s}=0.17$ denotes the Smagorinsky constant. Differently from Ries et al. [69] the usage of the ESF method is able to consider temperature fluctuations at the subgrid level for both, resolved and subgrid contributions.

Similarly, the entropy production through heat transfer is expressed as [69,70]

(15) $${\bar{\Pi }}_{Q}=\underset{\Pi_{Q}^{res}}{\underset{︸}{\left(\tilde{\frac{1}{T^{2}}}\right)\bar{\lambda }\frac{\partial \tilde{T}}{\partial x_{i}}\frac{\partial \tilde{T}}{\partial x_{i}}}}+\underset{\Pi_{Q}^{sgs}}{\underset{︸}{\frac{1}{2}\left(\tilde{\frac{1}{T^{2}}}\right)\bar{\rho }{\bar{c}}_{p}\varepsilon_{\sigma_{T,sgs}^{2}}}},$$

where $\varepsilon_{\sigma_{T,sgs}^{2}}$ stands for the dissipation rate of the subgrid-scale temperature variance, which can be formulated using the Obukhov-Corrsin inertial-convective subrange [71] scaling as

(16) $$\varepsilon_{\sigma_{T,sgs}^{2}}=\frac{4\pi^{2/3}Pr^{1/2}\nu_{sgs}}{3C_{OC}C_{S}^{4/3}\Delta^{2}}\sigma_{T,sgs}^{2}.$$

Here, $C_{OC}=1.34$ denotes the Obukhov-Corsin constant. Differently from previous approaches [69,70], which rely on additional closures to represent $\sigma_{T,sgs}^{2}$ based on resolved quantities, the subgrid scale temperature variance is readily available from the filtered PDF approximated through the Eulerian stochastic fields.

Going over to the entropy production through mixing, the decomposition yields

(17) $${\bar{\Pi }}_{D}=\underset{\Pi_{D}^{res}}{\underset{︸}{\frac{\bar{\lambda }}{{\bar{c}}_{p}}\sum_{k}^{N}\frac{R_{k}}{{\tilde{Y}}_{k}}\frac{\partial {\tilde{Y}}_{k}}{\partial x_{i}}\frac{\partial {\tilde{Y}}_{k}}{\partial x_{i}}}}+\underset{\Pi_{D}^{sgs}}{\underset{︸}{\frac{1}{2}\bar{\rho }\sum_{k}^{N}\frac{R_{k}}{{\tilde{Y}}_{k}}\varepsilon_{\sigma_{Y_{k},sgs}^{2}}}}.$$

In analogy to Equations (15) and (16), the dissipation rate of the species mass fraction variance is computed as

(18) $$\varepsilon_{\sigma_{Y_{k},sgs}^{2}}=\frac{4\pi^{2/3}Sc^{1/2}\nu_{sgs}}{3C_{OC}C_{S}^{4/3}\Delta^{2}}\sigma_{Y_{k},sgs}^{2}.$$

Similarly to [15,18], the entropy source term through chemical reaction is computed at runtime through integration in composition space and does not necessitate any subgrid modeling.

## 3. Configuration and Numerical Setup

The present section introduces the experimental configuration investigated and specifies the numerical setup based on the previously introduced methods.

### 3.1. Experimental Configuration

**Figure 1:**
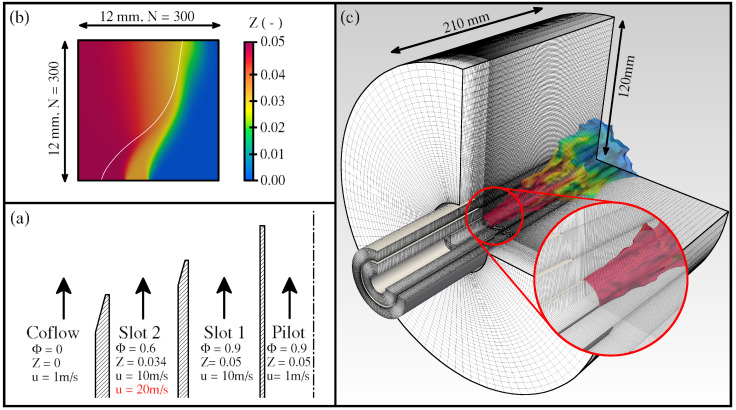
(**a**) Burner schematics of the Darmstadt Stratified Burner with operating conditions in the respective streams. (**b**) Computational domain and mixture fraction field for the simplified detailed chemistry computations performed in Section 4.1. The white line is a $Y_{\begin{array}{c} errortypeceCO2 \end{array}}=0.05$ isoline indicating the flame position. (**c**) The computational domain used for the simulations of the turbulent flames presented in Section 4.1. The flame position is illustrated by the isosurface T=1500K colored by the mixture fraction (for TSF-A-r).

The considered configuration is the Darmstadt Stratified Burner initially introduced by Seffrin et al. [24]. The burner was designed to deliver extensive validation data for numerical investigations of turbulent stratified combustion under lean conditions [24,25,30,72]. The burner consists of three concentric tubes, yielding three streams and a coflow. The flame is stabilized by a flame holder placed inside the inner tube, which is denoted as the pilot stream in Figure 1a. The configuration allows to vary the reactants mixture, which consists of $\begin{array}{c} {\mathrm{CH}}^{4} \end{array}$-air mixtures, and flow conditions in the two annular slots surrounding the pilot and by that, to set different levels of stratification or shear between the streams. To minimize heat losses, the pilot tube is made of sintered ceramic. A detailed description of the burner is provided in [24].

In this work, two operating conditions of the Darmstadt Stratified Burner are investigated, namely the TSF-A-r and the TSF-D-r configurations. The mixture and flow conditions are depicted in Figure 1a. Both cases feature the same level of stratification, which is realized by varying the equivalence ratio between slot 1 (equivalence ratio ϕ=0.9) and slot 2 (ϕ=0.6). However, the velocity in slot 2 is twice as high for TSF-D-r ( 20 m/s), yielding a Reynolds number Re = 26,600 for this slot and case (10m/s and Re = 13,300 for TSF-A-r). The Reynolds number in slot 1 is for both cases 13,800, based on a bulk velocity of 10 m/s. A minimum tube length of 25 hydraulic diameters implies a fully developed turbulent flow in both streams [24].

### 3.2. Numerical Setup

As outlined in the introduction section of this paper, one goal is to show that the tabulated chemistry approach is appropriate to compute the various entropy source terms introduced in Section 2.3. For this purpose, first, a simplified laminar version of the Darmstadt stratified burner is computed. The quadratic two-dimensional computational domain is shown in Figure 1b and consists of 300 × 300 control volumes. To ensure the stabilization of the slower laminar flame, the velocities of slot 1 and slot 2 are reduced to 0.77m/s and 0.58m/s, respectively. For this study, three approaches are applied and set aside. These are depicted in Table 1. The first approach consists in solving the full set of species transport equations, including the detailed reaction kinetics which are treated using the well-established GRI-3.0 reaction mechanism [73]. Note that the diffusion coefficient is obtained by applying a unity Lewis number to all species. The entropy source terms are then computed based on this solution and are referenced subsequently as $\Pi_{i,DC}$. At the same time, the fields obtained can be used to perform an *a priori* evaluation of the chemistry tabulation approach. As previously pointed out, the lookup table consists of one-dimensional premixed flamelets assuming a Lewis number of unity for all species. The second procedure consists in reconstructing the mixture fraction and progress variable from the detailed chemistry simulation and using these to obtain the thermochemical state stored in the table. This state can then be employed to compute the entropy production terms, which are denoted as $\Pi_{i,TAB,prio}$. The third approach is to transport the mixture fraction and progress variable directly and to evaluate the sources of entropy *a posteriori*, therefore termed $\Pi_{i,TAB,post}$. Note that, as the case is laminar, no turbulence-chemistry interaction (TCI) model is required for this case.

The computational domain used for the turbulent flames TSF-A-r and TSF-D-r is shown in Figure 1c. The cylindrical domain consists of 5.5 million hexahedral control volumes and ranges 180 mm downstream of the burner exit plane. In order to reproduce the turbulent flow in both slots, 120 mm of the burner upstream geometry is included in the computational domain. The fully developed turbulent flow is then achieved through a recycling method. A laminar flow is prescribed for the pilot and coflow. Since the flame holder is not included in the computational domain, the mixture at the pilot inlet is set to a fully burnt state and the velocity is adjusted to conserve the mass flux. All other boundaries are set to total pressure boundary conditions to enable the entrainment of the surroundings. The near-wall region is modeled using the wall function by Spalding [74]. The σ-eddy-viscosity model [36] is applied to model the subgrid momentum fluxes. To access the impact of the number of stochastic fields on the simulation results, each configuration is computed using 4, 8 and 16 stochastic fields. The investigations are performed using the open-source code OpenFOAM [75]. Here, a merged PISO [76]—SIMPLE [77] algorithm is applied to solve the system of partial differential equations [78]. Regarding the temporal discretization, a second-order implicit backward time-stepping scheme is applied to all fields except the stochastic fields, for which a first-order implicit Euler scheme has been used due to the stochastic nature of the equations. The convective momentum fluxes are discretized using a blended scheme with filtering of high-frequency modes [79]. The convective scalar fluxes are treated using the Minmod flux-limiter scheme [80]. A summary of the overall solution procedure is given in [54].

## 4. Results

In this section, the main results are summarized. The section is divided into two parts. The first part is dedicated to the simplified laminar configuration introduced in the previous section. Thereafter, the results obtained for the two operating conditions of the Darmstadt stratified burner are presented.

### 4.1. Entropy Generation in a Laminar Stratified Flame

**Figure 2:**
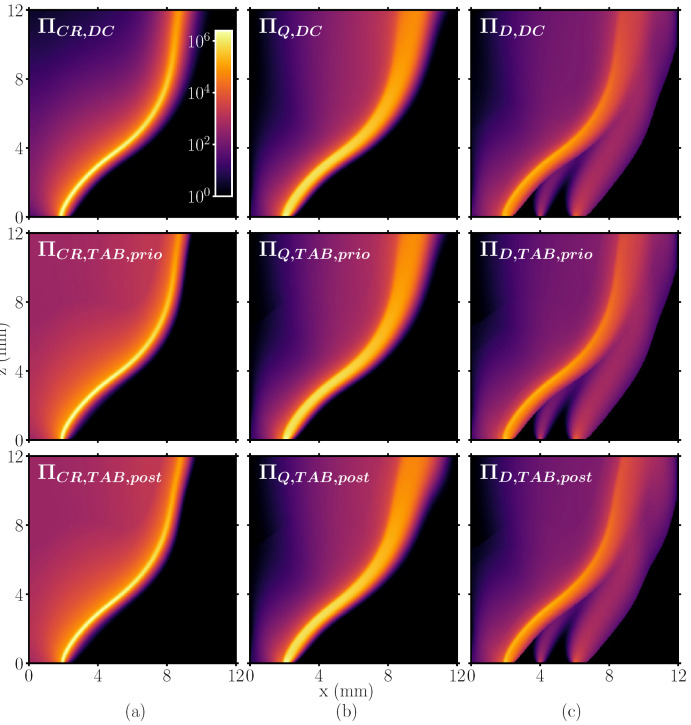
Comparison of the three methods to compute the entropy production terms (see Table 1) for (**a**) $\Pi_{CR}$, (**b**) $\Pi_{Q}$ and (**c**) $\Pi_{D}$.

Before going over to investigating the various sources of entropy in turbulent flames, it must be shown that the tabulated chemistry framework applied can indeed reproduce the results obtained when solving the reaction kinetics and scalar transport directly. This is not necessarily the case since the adopted tabulation strategy relies on one-dimensional premixed flamelets, thus neglecting any cross-flamelet interaction. For this purpose, the previously introduced approaches are compared for the simplified configuration shown in Figure 1b. This is first performed qualitatively in Figure 2, where the entropy source terms through chemical reactions, heat transfer and mixing are shown.

The first row corresponds to the results obtained using the detailed chemistry solution $\Pi_{i,DC}$, which is the reference for the two other approaches. The second row illustrates the entropy source terms based on the table lookup, where the control variables have been obtained from the detailed chemistry solution ($\Pi_{i,TAB,prio}$). In the third row, the solution is completely decoupled from the detailed chemistry approach and fully based on the tabulated approach ($\Pi_{i,TAB,post}$). Looking at the detailed chemistry results in the upper row of Figure 2, it can be observed that the source terms by chemical reaction and heat transfer dominate. However, $\Pi_{CR}$ is much narrower than $\Pi_{Q}$. This is because chemical reactions predominantly occur in a thin reaction zone, while the temperature gradients linked to $\Pi_{Q}$ are present in a broader region. In the case of the mixing term $\Pi_{D}$, three regions of entropy production can be noticed at lower axial positions, which strongly vary in their magnitude. The most inner region is attributed to the combustion reaction and the sharp jump in the species profiles. Even though the overall magnitude is much smaller than $\Pi_{CR}$ and $\Pi_{Q}$, it still appears several orders of magnitude greater than the entropy production in the two other regions (located further outside). These regions of higher entropy generation through mixing are associated with the stratification, i.e. the mixing between slot 1 and slot 2, and slot 2 and the coflow.

A first qualitative comparison between the three approaches suggests that all approaches are able to reproduce the detailed chemistry results. Nonetheless, it can be observed that the source term through chemical reaction is not decreasing as quickly behind the flame front for both $\Pi_{CR,TAB,prio}$ and $\Pi_{CR,TAB,post}$. One reason might be the known difficulty to properly resolve the entire thermochemical state in the vicinity of the chemical equilibrium using a single progress variable [81,82]. However, it must be noted that the source term value in this region is still several orders of magnitude below its maximum value.

**Figure 3:**
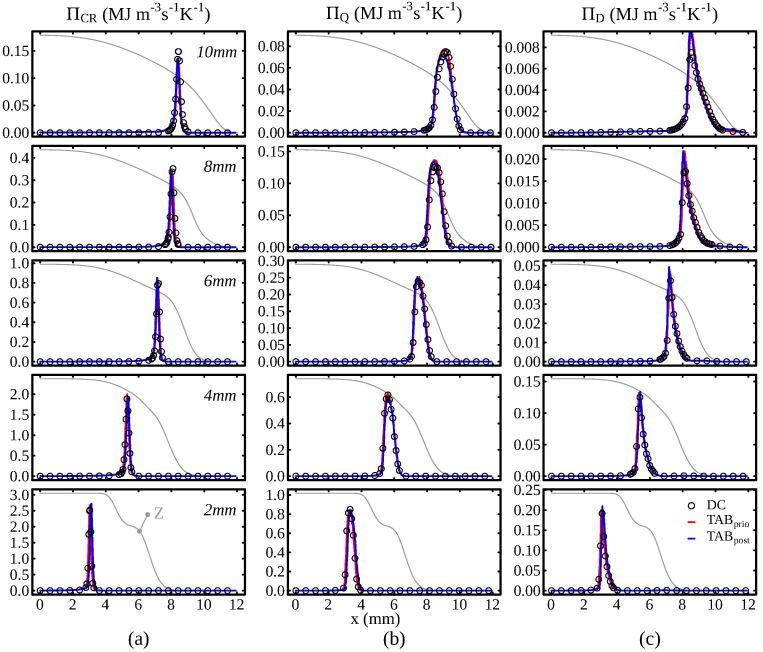
Comparison of entropy source terms by (**a**) chemical reaction, (**b**) heat transfer and (**c**) mixing at different axial positions for the simplified two-dimensional stratified flame. The gray line shows the mixture fraction profile qualitatively at the respective positions.

Next, a quantitative comparison of the results is provided in Figure 3. The figure shows the profiles of the various entropy sources along the *x*-axis at several distances (*z*-axis) from the burner exit. The previous observations are confirmed: (1) While the peak value of $\Pi_{CR}$ is higher than for the other contributions, the entropy is only produced in a very narrow region, which agrees well with the findings of [9] in one-dimensional flames. (2) The main contribution to $\Pi_{D}$ stems from the chemical reaction, not the stratification. In addition to the entropy source terms, the mixture fraction profile is shown qualitatively in the plots. It can be observed that downstream of z=4mm the flame burns in a stratified regime. However, the strongest mixture fraction gradients interact with the flame further downstream. These are also the only positions where slight deviations can be perceived between the tabulated approaches and the detailed chemistry simulation. The reason for these deviations is that minor species are generally hard to predict with tabulated approaches in the presence of mixture fraction gradients [81,83]. Nonetheless, this aspect has apparently only a marginal influence on the overall entropy generation. It can be concluded that the tabulation strategy can access the entropy production qualitatively and quantitatively for the configurations of interest.

### 4.2. Entropy Generation in Turbulent Stratified Flames

The present section is dedicated to the two turbulent flames TSF-A-r and TSF-D-r. First, a validation of the modeling framework is presented through comparison with experimental measurements. Thereafter, the flames are characterized with special focus on the entropy generation.

#### 4.2.1. Comparison with Experimental Data

In this section, an overview of the obtained numerical results and a validation of the framework as a whole are provided. First, the general characteristics of the flames are briefly discussed based on instantaneous snapshots of the flame. Thereafter, an extensive quantitative comparison with experimental data is presented to demonstrate the validity of the approach.

**Figure 4:**
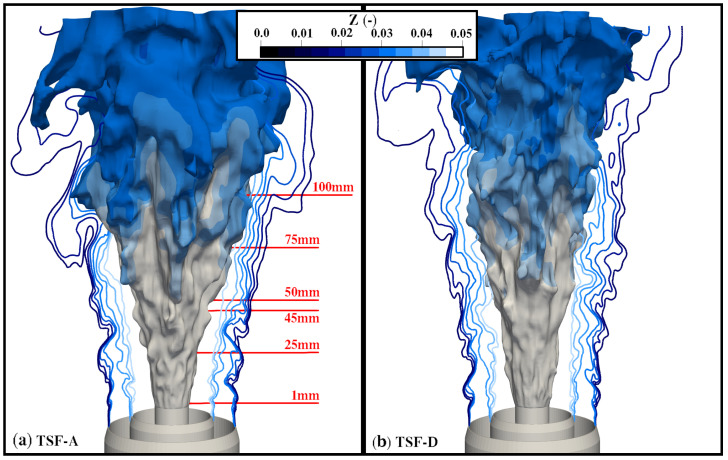
Temperature isosurface T=1500K colored by mixture fraction alongside mixture fraction isolines illustrating the stratification between the different streams for (**a**) TSF-A-r and (**b**) TSF-D-r. The axial positions at which simulation data are quantitatively compared to experiments are shown in red.

A first impression of the investigated flames is given in Figure 4. It can be observed that the TSF-D flame is much narrower, suggesting that the higher velocity in slot-2 pushes the flame inward, which is in agreement with photographs shown in [24].

**Figure 5:**
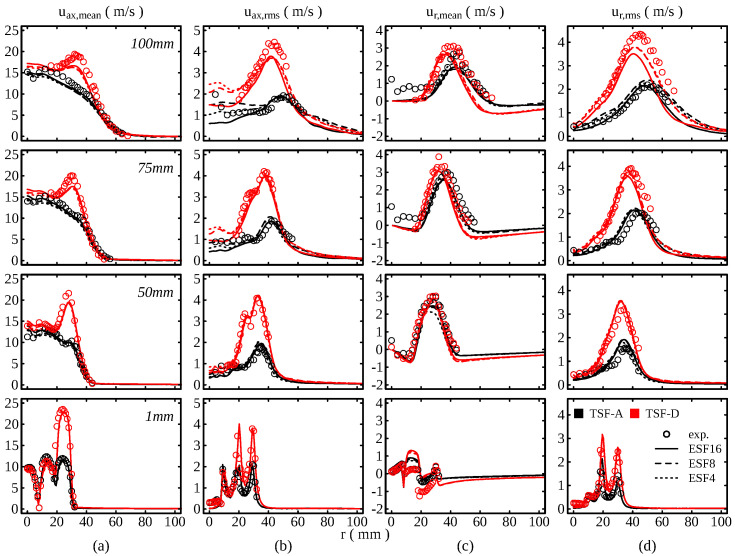
Comparison of the flow field temporal statistics with experimental data. (**a**) Mean axial velocity (**b**) rms axial velocity (**c**) mean radial velocity (**d**) rms radial velocity.

Going over to the quantitative comparison, Figure 5 shows the temporal statistics of the flow field at several axial positions downstream of the burner exit (for reference, the positions are depicted in red in Figure 4a). At the lowest position z=1mm, three distinct peaks for the axial velocity mean can be observed, corresponding to the different burner streams. The respective velocities in the pilot, slot 1 and slot 2 agree well with the experimental measurements for both TSF-A-r and TSF-D-r. This behaves similarly for the rms values of the velocity field. Moreover, the simulations correctly reflect the higher velocity in slot 2, which induces higher shear and higher velocity rms values. Moving to higher axial distances from the burner exit, the simulation results are able to reproduce the experimental measurements with great consistency. The previous observation of a reduced spreading of the flame is confirmed by the velocity measurement. The peak in the radial velocity mean and rms is caused by the presence of the flame and it can be observed that this peak moves to higher radial distances for TSF-A-r. In contrast, the flame remains narrower for TSF-D-r. This effect is well captured in the simulations.

The results suggest that mean quantities are almost not affected by the number of stochastic fields used. However, the rms values appear to be more sensitive to the number of fields used. This becomes apparent at higher distances from the burner exit and could be caused by the low number of stochastic fields, for which the stochastic contributions tend to increase the fluctuations artificially. The profiles are consistent with the results by Avdic et al. [37].

**Figure 6:**
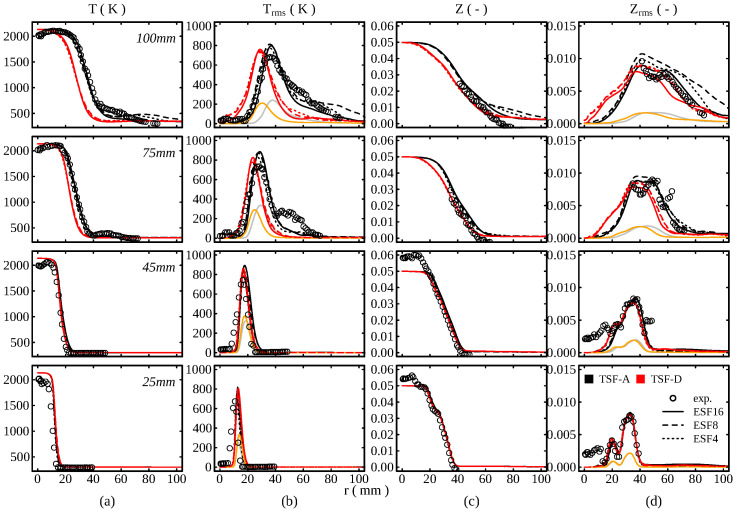
Comparison of the scalar temporal statistics with experimental data. (**a**) Mean temperature, (**b**) rms temperature, (**c**) mixture fraction mean, (**d**) mixture fraction rms. The gray (TSF-A) and orange (TSF-D) lines in (**b**,**d**) correspond to the subgrid contribution to the total rms values (only results using 16 stochastic fields are shown).

In order to compute the various entropy sources in these configurations, it is also crucial to reproduce the experimental scalar fields correctly. The temporal statistics of temperature and mixture fraction are therefore compared to experimental measurements in Figure 6. Note that experimental data is only available for TSF-A. A general observation is that the simulations are able to reproduce the main characteristics observed in the experiments for both temperature and mixture fraction. However, some differences are noticeable, for instance, the higher temperature at the centerline at lower axial positions. This overprediction of the temperature comes from the adiabatic assumption made in the lookup-table generation and has also been observed in [25,27]. Even though the experimental setup strives to minimize heat losses, these are clearly present. This yields a slight shift towards higher radii for the average temperature increase and flame position. The observed deviations decrease further downstream and the temperature mean is in good agreement with the experiment. The flame dynamics appear to be well captured by the simulation, which is indicated by the good agreement in the temperature rms, where the peak values are marginally overpredicted by the simulation. Looking first at the mixture fraction temporal average profiles, the different equivalence ratios in the streams are still noticeable at the lowest axial positions z=25mm. As axial distance increases, these steps merge into one continuous profile. Similar to the temperature profiles, even though the experimental trends are well reproduced, the simulation shows some deviations from the experiments. However, considering the experimental uncertainty, which can be estimated from the mismatch of experiment and simulation at the centerline at the two lowest axial positions, these discrepancies appear reasonable. Differently, the mixture fraction rms matches the experimental measurements in shape and magnitude. As observed for the flow field statistics, the results show no strong sensitivity on the number of stochastic fields used.

In summary, the simulation results are able to reproduce the main characteristics of the configurations reasonably well, which justifies the further investigations presented in the next section.

#### 4.2.2. Entropy Production Analysis

**Figure 7:**
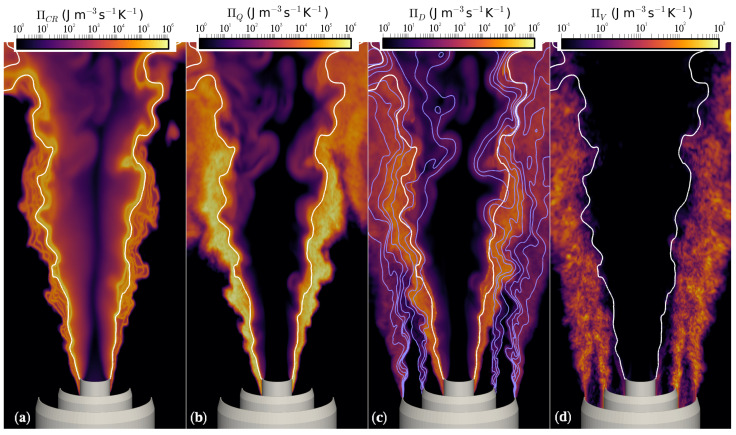
Instantaneous contours of (**a**) $\Pi_{CR}$ (**b**) $\Pi_{Q}$ (**c**) $\Pi_{D}$ (**d**) $\Pi_{V}$for TSF-D-r. The position of the flame is represented by the white progress variable isoline (PV=0.001), whereas the stratification is illustrated by the purple mixture fraction isolines in (**c**). Note the different scaling for $\Pi_{V}$.

The present section investigates the different entropy production terms for the turbulent stratified flames investigated. For this analysis, the most detailed simulations are used, i.e., the simulations with 16 stochastic fields. To get an initial impression of the different contributions, instantaneous contours of $\Pi_{CR}$, $\Pi_{Q}$, $\Pi_{D}$ and $\Pi_{V}$ are shown for TSF-D-r in Figure 7. TSF-A-r is for now omitted from the analysis but is included in the quantitative investigations shown later in this section. To get an idea of the flame position and stratification, *Z* and PV=0.001 isolines are included in the contour plots. Starting with the source term by chemical reaction, it can be observed that the main part is located in direct proximity of the PV isoline. Another interesting point is that $\Pi_{CR}$ broadens with increasing axial distance from the burner exit. This is on one hand caused by the flame burning in leaner conditions, resulting in an increased flame thickness. On the other hand, the coarser resolution and the increased subgrid modeling at higher axial positions yield a stronger stochastic contribution to the stochastic fields transport equations. This facilitates their distancing from the mean. In contrast to the chemical reaction source term, the entropy production through heat exchange $\Pi_{Q}$ appears most significant in regions located in front of the reaction zone, i.e., in the preheating zone. At first glance, exergy losses through heat transfer seem to dominate for this type of flame. With respect to $\Pi_{D}$, as for the laminar case, three regions can be observed. While the two outer regions correspond to the entropy source emerging from the stratification layers between the different streams, the inner region can be attributed to mixing over the flame front. Apparently, the contribution to $\Pi_{D}$ emerging from the species diffusion over the flame front dominates. This is due to the higher species gradients across the flame front when compared to the mixing taking place in the stratification layers. The total contribution of $\Pi_{D}$ appears small compared to the two previously discussed sources of entropy. Finally, for $\Pi_{V}$, it can be observed that the main contributions arise between the different streams, i.e., in the shear layers. Moreover, it can be observed that this term is almost zero behind the flame, which agrees well with the physics expected at this position. Behind the flame front, the turbulence level tends to naturally reduces, which is due to an increased viscosity that goes along with the temperature rise. The observation is also in a agreement with the low centerline velocity fluctuations noticed in Figure 5. Compared to the other contributions, viscous dissipation plays a minor role, which agrees well with previous findings [9,12,18].

**Figure 8:**
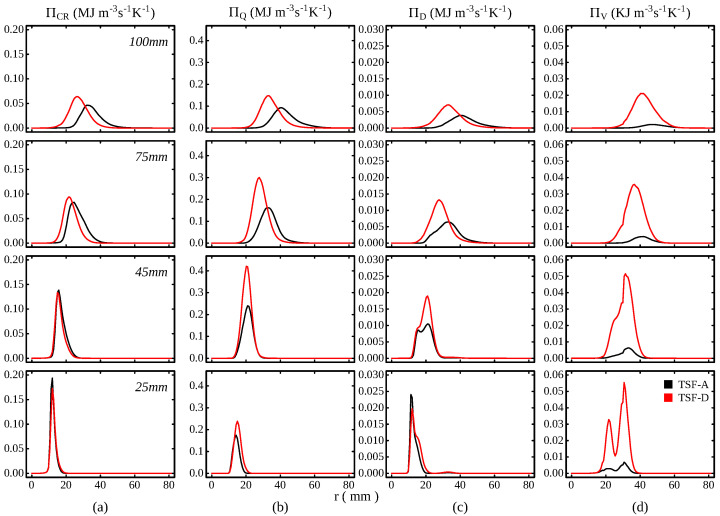
Radial profile of the time averaged entropy production terms at various positions downstream of the burner exit. (**a**) $\Pi_{CR}$ (**b**) $\Pi_{Q}$ (**c**) $\Pi_{D}$ (**d**) $\Pi_{V}$.

Next, radial profiles of the time-averaged entropy production terms are shown in Figure 8 for TSF-A-r and TSF-D-r, respectively, at four positions downstream of the burner exit plane. At the lowest axial positions, the profiles obtained in the simulation are similar. This is because, the impact of the higher bulk velocity in slot 2 has almost no influence on the entropy production terms. However, the entropy production through viscous dissipation is considerably larger for TSF-D-r. This can be consistently observed at all distances from the burner exit due to the higher Reynolds number in slot 2 and the stronger shear between the burner streams. Nevertheless, as previously outlined, the overall entropy production through viscous effects is still at least two orders of magnitude smaller than the other contributions. Further downstream, the impact of the higher bulk velocity and turbulence in slot 2 becomes apparent in the profiles of $\Pi_{Q}$ and $\Pi_{D}$. At z=4545mm, the overall entropy production through mixing and heat transfer are almost doubled for TSF-D, while $\Pi_{CR}$ remains almost unaffected. The explanation for this is that the entropy production through chemical reaction occurs in a rather narrow range, whereas $\Pi_{Q}$ and $\Pi_{D}$ are distributed more broadly (and slightly shifted towards the fresh gas mixture, which is more pronounced for $\Pi_{Q}$). These terms are therefore likely to be influenced earlier by the stronger turbulence in the outer slot. The impact on the chemical source term becomes visible as the distance from the burner further increases. At these positions, the profiles differ not only in the position of the peak (due to the different flame positions) but also in their maximum values, which are slightly higher for TSF-D-r. The broadening of the peak downstream of the burner exit results from the turbulent flame brush, which consequently reduces the maximum values of $Pi_{Q}$, $Pi_{D}$ and $Pi_{CR}$. The results suggest that the increased shear induced by a stronger bulk velocity has not such a strong influence on the entropy production through chemical reactions as is the case for the other terms. To evaluate the overall impact of the increased bulk flow in slot 2, the relative contributions to the total entropy production are presented in Table 2 for both operating conditions. Note that due to the particular burner configuration, i.e., that the flame does not resemble a classical jet flame, entropy production is still expected outside of the computational domain. Nonetheless, the present results can at least give an estimation of the impact of a stronger shear in the near burner region. The results suggest that the higher shear yields higher entropy production through heat, mixing and viscous dissipation and reduces the fraction by chemical reaction. These observations are consistent with the flame regime characterization of the flame TSF-A-r provided by Kuenne et al. [25]. According to the authors, the flame is expected to burn in the *thickened-wrinkled* flame zone [84,85]. This indicates that the smallest turbulent scales are expected to enter the flame and interact with its structure. However, as pointed out by Poinsot and Veynante [86], the influence of these small scales is limited to the preheating zone for Karlovitz number up to 100, which holds true for the flames investigated. This is likely to be the reason for the minor impact of the increased bulk flow on the entropy production through chemical reaction. On the other side, the significant increase in the entropy production through heat transfer can be explained by a stronger interaction of turbulent structures with the flame preheating zone.

Finally, a brief discussion regarding the impact of the adopted adiabatic tabulation strategy is provided. Based on the observations made by Kuenne et al. [87], the adiabatic assumption has two main effects: (1) As we have stated in our manuscript, neglecting heat losses to the burner walls yields higher temperatures at the centerline downstream (above the pilot) of the burner exit. However, a meaningful impact on the entropy sources is not expected. This is because no strong temperature, species, or velocity gradients are present in this region, which is indicated by the low temporal variances observed at theses positions. (2) In direct proximity of the burner, the heat losses to the burner wall will on one hand yield higher (wall-normal) temperature gradients close to the walls and is thus likely to increase $\Pi_{Q}$. On the other hand, the heat losses will reduce chemical reaction and eventually cause flame quenching/lift-off, yielding lower $\Pi_{CR}$ and $\Pi_{D}$ in direct proximity of the burner exit.

## 5. Conclusions

For the first time, a straightforward strategy to investigate entropy production in premixed combustion, more precisely in stratified premixed flames, was presented. The modeling approach relied on a chemistry tabulation strategy to represent the detailed chemistry at low computational costs, which is crucial to compute the various sources of exergy losses in combustion systems. The important outcomes can be summarized as follows: The performance of the approach was first evaluated for a simplified stratified premixed flame, where it was compared to results using a detailed representation of the chemistry for the reaction kinetics and transport *a priori* and *a posteriori*. It was shown that the tabulated chemistry approach is well suited to compute the various contributions to the exergy losses, even though slight deviations could be observed for the mixing term in regions of strong stratification. Second, the tabulated chemistry approach was applied in the context of LES to compute the Darmstadt stratified burner. The Eulerian stochastic field method was used to represent the turbulence chemistry interaction which, in turn, was used to provide a closure for the filtered entropy source terms. Third, the effects of the operating conditions on the entropy production have been pointed out. For this purpose, two operating conditions of the Darmstadt stratified burner with varying levels of shear have been considered.

- Through comparison with available experimental data it was demonstrated that the approach is able to represent the temporal flow field and scalar field statistics reasonably well for both operating conditions.
- From an analysis of the entropy production, qualitatively and quantitatively, the characterization of various entropy generation sources was achieved. It turned out that the main contribution is from heat transfer, followed by the chemical reaction.
- It was shown that the higher shear in slot 2 for TSF-D-r yields higher entropy production through heat, mixing and viscous dissipation and reduces the share by chemical reaction to the total entropy generated. The largest differences could be observed for the source term by heat transfer and the underlying phenomena could be identified, namely the stronger interaction of turbulent structures with the flame preheating zone.

The present contribution combines a detailed representation of the chemistry with an advanced subgrid closure for the filtered entropy production and clearly shows potential for future second-law-based investigations in turbulent premixed flames in technical systems, providing the foundation for detailed entropy-based optimizations. To further improve the understanding of exergy analysis and its modeling within combustion applications, the impact of the simplifications in the molecular diffusion representation (linked to the IEM model) should be examined, for instance through comparisons with results achieved by using more sophisticated micro-mixing models. While this aspect is beyond the scope of this paper, it offers room for future investigation.

## Figures and Tables

**Table 1:** Overview of the approaches applied to compute the entropy production terms for the simplified laminar version of the Darmstadt stratified burner.

Approach	Transported Quantities	Computation of $\Pi_{i}$ via
*DC*	$Y_{k}$, *h*	transported $Y_{k}$
*TAB,prio*	$Y_{k}$, *h*	tabulated $Y_{k}$
*TAB,post*	*Z*, PV	tabulated $Y_{k}$

**Table 2:** Relative contributions to the total entropy production in the computational domain $\int \Pi_{i}dV/\int \sum_{i}\Pi_{i}dV$ for the two operating conditions considered.

	TSF-A-r	TSF-D-r
$\Pi_{Q}$	69.557%	71.780%
$\Pi_{CR}$	26.523%	23.935%
$\Pi_{D}$	3.917%	4.262%
$\Pi_{V}$	0.004%	0.023%

## Data Availability

Not applicable.

## Abbreviations

CR	Chemical reaction
DC	Detailed chemistry
EMST	Euclidean minimum spanning tree
ESF	Eulerian stochastic fields
FDF	Filtered PDF
FP	Fokker-Planck
IEM	Interaction by exchange with the mean
LES	Large eddy simulation
LMSE	Linear mean square estimation
PDF	Probability density function
RHS	Right-hand side
sgs	Subgrid-scale
TAB	Tabulated
TCI	Turbulence-chemistry interaction
